# Impact of Concomitant 5-Aminosalicylic Acid Therapy on Vedolizumab Efficacy and Safety in Inflammatory Bowel Disease: *Post Hoc* Analyses of Clinical Trial Data

**DOI:** 10.1093/ecco-jcc/jjad113

**Published:** 2023-07-26

**Authors:** Ryan C Ungaro, Harisha Kadali, Wenwen Zhang, Shashi Adsul, Walter Reinisch

**Affiliations:** The Henry D. Janowitz Division of Gastroenterology, Icahn School of Medicine at Mount Sinai, New York, NY, USA; Takeda, Global Patient Safety and Evaluation, Cambridge, MA, USA; Takeda, Statistical and Quantitative Sciences, Cambridge, MA, USA; Takeda, Global Medical Affairs, Cambridge, MA, USA; Medical University of Vienna, Department of Internal Medicine III, Vienna, Austria

**Keywords:** Clinical trials, endoscopy, biomarkers

## Abstract

**Background and Aims:**

The benefit of continuing 5-aminosalicylic acid [5-ASA] treatment when escalating to advanced therapies in patients with inflammatory bowel disease [IBD] is unclear. Vedolizumab is a gut-selective monoclonal anti-α_4_β_7_-integrin antibody used to treat moderate to severe IBD. Clinical trial data were analysed *post hoc* to assess the impact of 5-ASA co-treatment on vedolizumab efficacy and safety in patients with IBD.

**Methods:**

Data were analysed from patients aged 18–80 years with moderate to severe ulcerative colitis [UC]/Crohn’s disease [CD] receiving intravenous [IV]/subcutaneous [SC] vedolizumab. Efficacy data were from four studies [GEMINI 1 and 2 and VISIBLE 1 and 2]; safety data were from seven studies [GEMINI 1‒3 and long-term, VISIBLE 1, 2, and open-label extension]. The impact of 5-ASA co-treatment on clinical and endoscopic outcomes at Weeks 6 and 52 was assessed using multivariate analysis (adjusted odds ratios [aORs] with 95% confidence intervals [CIs]).

**Results:**

There were no significant differences in UC clinical remission [Mayo score ≤2, no subscore >1] rates with vs without 5-ASA at Week 6 [20.7% vs 20.4%, respectively; aOR 0.77, 95% CI 0.43–1.38] or at Week 52 [45.1% vs 40.6%; aOR 1.14, 0.70–1.86], and in CD clinical remission [CD activity index score ≤150] rates at Week 6 [41.4% vs 35.1%; 1.26, 0.86–1.85] or at Week 52 [49.6% vs 37.8%; 1.35, 0.91–1.99]. The incidence of enteric and all infections in vedolizumab IV/SC-treated patients was low with and without 5-ASA.

**Conclusion:**

Continuation of concomitant oral 5-ASA after starting vedolizumab had no significant impact on clinical and endoscopic outcomes.

**Clinical trial identifiers:**

GEMINI 1: NCT00783718, EudraCT 2008-002782-32; GEMINI 2: NCT00783692, EudraCT 2008-00278-33; GEMINI 3: NCT01224171, EudraCT 2009-016488-12; GEMINI long-term safety study: NCT00790933, EudraCT 2008-002784-14; VISIBLE 1: NCT02611830, EudraCT 2015-000480-14; VISIBLE 2: NCT02611817, EudraCT 2015-000481-58; VISIBLE open-label extension: NCT02620046, EudraCT 2015-000482-31.

## Graphical Abstract

**Graphical Abstract:**
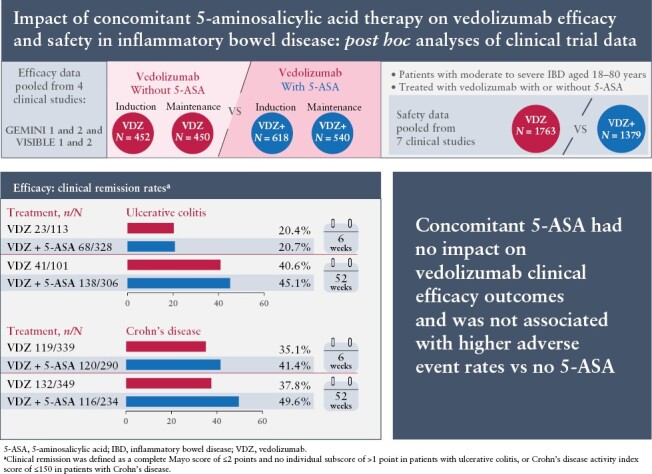

## 1. Introduction

5-Aminosalicylic acid [5-ASA]^1–5^ agents are more commonly used for the management of ulcerative colitis [UC] than Crohn’s disease [CD].^2,6,7^ Professional guidelines recommend 5-ASA therapy [oral and/or rectal] as a first-line treatment in mild to moderate UC.^7,8^ However, 5-ASA is not recommended for induction or maintenance of remission in mild to moderate CD,^9,10^ and there is an ongoing debate as to the benefit of 5-ASA in CD management.^9,11,12^ Despite recommendations and the limited data on effectiveness, patients with CD often receive 5-ASA treatment as monotherapy or in combination with other treatments.^2,11,13^ A retrospective observational study of the US Truven Health MarketScan health claims database identified 5-ASA monotherapy as the most common treatment during the first year of CD diagnosis, used by 39.3% of patients.^2^ In patients with UC or CD who do not respond to 5-ASA initially or who later lose response, advanced targeted therapies, such as biologics, are initiated, frequently as add-on treatments while oral 5-ASAs are continued.^13–16^

A possible reason for the continued use of 5-ASA in inflammatory bowel disease [IBD], even after failure of induction or maintenance therapy, may be due to perceived chemoprotective effects of 5-ASA treatment. This is based on some studies reporting reduction of colorectal cancer risk with 5-ASA treatment,^17–20^attributing this effect to the promotion of mucosal healing and/or unknown molecular effects on carcinogenesis.^17,21^*Post hoc* clinical trial analyses and studies of observational health claims data have so far not indicated a clear benefit for concomitant 5-ASA treatment in terms of clinical effectiveness and/or safety outcomes for patients escalated to anti-tumour necrosis factor-α [anti-TNFα] treatment.^12,16,22–28^ As the therapeutic armamentarium for IBD expands, it is important to understand if there is any benefit to continuing 5-ASA in patients escalated to other advanced therapies that have differing mechanisms of action.

Vedolizumab is a gut-selective anti-α_4_β_7_-integrin humanized monoclonal antibody used to treat patients with moderate to severe UC or CD via intravenous [IV] infusion for induction and maintenance therapy.^29,30^ The subcutaneous [SC] formulation of vedolizumab has also been approved as a maintenance therapy for moderately to severely active UC or CD in Europe and other countries.^30–32^ Patients receiving vedolizumab IV or SC in the GEMINI and VISIBLE phase 3 clinical studies achieved high rates of clinical response and durable clinical remission, with vedolizumab demonstrating a favourable safety profile.^33–36^ Patients in these studies were permitted to continue stable concomitant medications, including 5-ASA. Data on 5-ASA and vedolizumab combination treatment are limited, with one small retrospective cohort study reporting that concomitant 5-ASA in vedolizumab-treated patients with UC provided no additional clinical benefit.^23^ The aim of the current *post hoc* analysis of vedolizumab clinical trial data was to assess the impact of concomitant 5-ASA on the efficacy and safety of vedolizumab IV and SC in patients with moderate to severe UC or CD.

## 2. Methods

### 2.1. Study population and treatment groups

**Figure 1.**
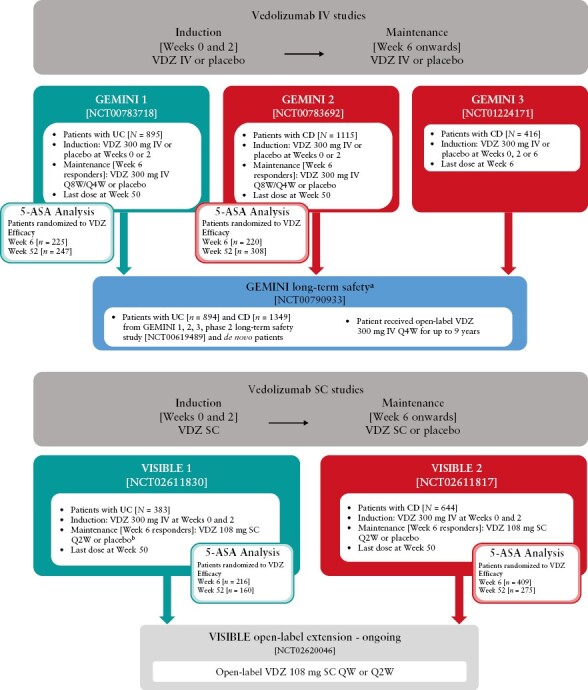
Summary of patient populations and treatment regimens in the studies included in the *post hoc* analysis. The *N* numbers represent the number of patients who were enrolled in each study. 5-ASA, 5-aminosalicylic acid; CD, Crohn’s disease, IV, intravenous; QW, once every week; Q2W, once every 2 weeks; Q4W, once every 4 weeks; Q8W, once every 8 weeks; SC, subcutaneous; UC, ulcerative colitis; VDZ, vedolizumab. ^a^Patients who previously took part in study C13004 [NCT00619489] and patients with moderate to severe CD or UC who were not previously treated with VDZ were also eligible to enrol. ^b^Induction: VDZ 300 mg IV at Weeks 0 and 2. Maintenance [Week 6 responders]: from Week 6, VDZ 108 mg SC or placebo Q2W for up to 52 weeks. In VISIBLE 1, patients were also randomized to VDZ 300 mg IV Q8W.

We conducted *post hoc* analyses of patient-level data from phase 3 clinical studies, five randomized, placebo-controlled trials [GEMINI 1, 2 and 3, and VISIBLE 1 and 2] and two non-randomized, open-label extension [OLE] studies [Figure 1]. GEMINI 1 [NCT00783718, EudraCT 2008-002782-32] and GEMINI 2 [NCT00783692, EudraCT 2008-00278-33] were 52-week studies of vedolizumab IV treatment^33–35^; GEMINI 3 [NCT01224171, EudraCT 2009-016488-12] was a 10-week vedolizumab IV study, predominantly in patients with CD refractory to anti-TNFα treatment^37^; and VISIBLE 1 and 2 were 52-week trials comprising vedolizumab IV induction and SC maintenance phases [VISIBLE 1: NCT02611830, EudraCT 2015-000480-14; VISIBLE 2: NCT02611817, EudraCT 2015-000481-58].^34,36^ The two OLE studies were the GEMINI long-term safety [LTS] study [NCT00790933, EudraCT 2008-002784-14], with patients receiving up to 9 years of vedolizumab IV treatment^38^; and VISIBLE OLE [NCT02620046, EudraCT 2015-000482-31], an ongoing long-term study of vedolizumab SC therapy.

These studies evaluated vedolizumab IV or SC treatment with or without concomitant corticosteroids [CS] or immunomodulators [IMMs] in patients with moderate to severe IBD who had an inadequate response, loss of response or intolerance to CS, IMM and/or anti-TNFα treatment. Only patients randomized to vedolizumab treatment for induction or maintenance therapy were evaluated. All patients included in the analysis were adults [aged 18–80 years] and had moderate to severe UC [GEMINI 1 and VISIBLE 1] defined as a Mayo score of 6–12 and endoscopic subscore of ≥2, with disease that extended proximal to the rectum [≥15 cm], or moderate to severe CD defined as a CD activity index [CDAI] score of 220–450 in GEMINI 2 and VISIBLE 2, and a CDAI score of 220–400 in GEMINI 3. Protocols for randomized studies permitted concomitant treatment with oral 5-ASA at a stable dose for the 2 weeks prior to study dosing and during the study. In addition, concomitant immunosuppressants [azathioprine or 6-mercaptopurine for UC; azathioprine, 6-mercaptopurine or methotrexate for CD] were permitted at a stable dose for 8 weeks prior to study dosing, and oral CS with stable dosing were permitted for 4 weeks prior to study dosing [or 2 weeks if CS therapy had just been initiated or was being tapered]. Stable CS dosing was maintained unaltered until Week 6 when patients were assessed using criteria for mandatory CS tapering [clinical response attained at Week 6 or at a subsequent study visit]. The tapering regimen was predefined according to CS treatment and dose. Prednisone doses >10 mg/day were reduced at a rate of 5 mg/week to 10 mg/day. Prednisone ≤10 mg/day and equivalent doses were reduced at a rate of 2.5 mg/week until discontinuation. Patients experiencing symptom recurrence on CS taper could have their CS dosage increased up to their baseline dose and were then to resume tapering within 2 weeks.

For all studies in this *post hoc* analysis, concomitant 5-ASA was defined as any dose of 5-ASA treatment [including any mesalamine or sulfasalazine formulation] received by the patient between the start and the end of their vedolizumab therapy. For efficacy outcomes, data from GEMINI 1 and 2 and VISIBLE 1 and 2 were assessed; for safety, data were assessed from the GEMINI 1, 2 and 3, VISIBLE 1 and 2, GEMINI LTS and VISIBLE OLE studies. All patients provided written informed consent before participation and the Institutional Review Board-approved studies were conducted and reported according to the study protocols.

### 2.2. Study endpoints

Efficacy and safety comparisons were made based on concomitant 5-ASA use for each indication [UC or CD].

#### 2.2.1. Efficacy evaluations

Efficacy outcomes included the proportion of patients achieving clinical remission at the end of induction [Week 6] and maintenance phase [Week 52], and CS-free remission at Week 52. Definition of clinical remission was a complete Mayo score of ≤2 and no individual subscore of >1 in patients with UC, and a CDAI score of ≤150 in patients with CD. CS-free remission was defined as patients who were receiving oral CS at baseline [Week 0] who had then discontinued oral CS and were in clinical remission at Week 52. Clinical response [defined as a reduction in complete Mayo score of ≥3 and ≥30% from baseline with an accompanying decrease in rectal bleeding subscore of ≥1 or absolute rectal bleeding subscore of ≤1 in patients with UC, and as a ≥100-point decrease in CDAI score from baseline in patients with CD] was also assessed.

Patients with normalization of faecal calprotectin [FCP], a biomarker of intestinal inflammatory activity, were defined as those with FCP ≤250 µg/g at Weeks 6 and 52. FCP data were only collected at baseline in the GEMINI 2 study. FCP normalization was evaluated in 275 patients with CD treated with vedolizumab SC in the VISIBLE 2 study only and reported in the manuscript on this study^36^ [insufficient data were available on endoscopy in any CD trials to allow robust analyses]. Multivariate analyses were conducted for pooled efficacy outcome data from patients who were treated with vedolizumab [IV or SC] during both induction and maintenance phases in GEMINI 1 and 2, and in the VISIBLE 1 and 2 studies. GEMINI 3 was not included in the efficacy analysis because it was an induction-only study. The proportion of patients achieving a clinical response at Week 52 was included in the multivariate analysis of efficacy for patients with CD for the purpose of corroborating findings on clinical remission at this time point. Odds ratios [ORs] for the outcomes of clinical response, clinical remission and CS-free clinical remission were adjusted *a priori* for age, sex, concomitant CS use at baseline [dose of prednisone], baseline IMM use, disease location, smoking status, baseline albumin, baseline FCP, prior anti-TNFα treatment experience and baseline Mayo endoscopy score [UC only].

#### 2.2.2. Safety evaluations

Safety data were derived from the assessment of adverse events [AEs] and serious AEs conducted per protocol for each study. AEs were coded according to the *Medical Dictionary for Regulatory Activities*. Safety data from the vedolizumab IV and SC studies were not combined because more recent *Medical Dictionary for Regulatory Activities* versions were used in the SC studies, precluding reliable pooling of data. AEs of special interest were predefined as gastrointestinal [GI] events. Incidence rates of all infections and GI infections were analysed separately. Safety data were pooled into two separate groups based on route of vedolizumab administration from the four clinical trials that assessed vedolizumab IV treatment [GEMINI 1, GEMINI 2, GEMINI 3 and GEMINI LTS] and from three clinical trials that assessed vedolizumab SC [VISIBLE 1, VISIBLE 2 and VISIBLE OLE]. AE data were reported after the first IV or SC dose of vedolizumab and within 126 days of the last IV or SC dose.

Safety data were also analysed in subgroups of patients receiving concomitant CS or IMM treatment with vedolizumab IV [data from GEMINI 1, 2 and 3 and GEMINI LTS] or with vedolizumab SC [using data collected in the maintenance phases of VISIBLE 1 and 2 and also for patients previously enrolled in VISIBLE 1 or 2 treated in the VISIBLE OLE study]. In addition, safety data were analysed by disease location in patients with CD [colon only, ileum only or ileocolonic] treated with vedolizumab IV or SC. Data for analysis by disease location in patients with CD receiving vedolizumab IV treatment were from three studies [GEMINI 2, GEMINI 3 and GEMINI LTS] and SC treatment data were from the maintenance phase of VISIBLE 2 and patients enrolled from VISIBLE 2 treated in the extension study [VISIBLE OLE].

### 2.3. Statistical analyses

Descriptive statistics were used to describe the baseline characteristics of patients treated with concomitant 5-ASA vs those without concomitant 5-ASA at the time of vedolizumab treatment initiation. A multivariate analysis was conducted for each efficacy outcome at each time point using logistic regression modelling with concomitant 5-ASA use, sex, baseline IMM use, disease location, smoking status, prior anti-TNFα treatment experience and baseline FCP categorization [≤250 vs >250 µg/g] as fixed categorical factors, and age, concomitant CS use at baseline [dose of prednisone], baseline albumin and baseline Mayo endoscopy score [UC only] as numerical covariates. Adjusted ORs [aORs; with 5-ASA vs without 5-ASA] and the associated 95% confidence intervals [CIs] were reported for each outcome. Patients with missing data were imputed as non-responders or non-remitters. For the analysis of safety data, proportions of patients with adverse events were reported. In addition, exposure-adjusted incidence rates (per 100 patient-years [PY]) were reported for infections and enteric infections.

## 3. Results

### 3.1. Study population

Data from seven clinical trials of vedolizumab-treated patients with moderately to severely active IBD were included in the *post hoc* analysis. The analysis of efficacy data [from four studies] included 1070 vedolizumab-treated patients from the induction phase [441 with UC and 629 with CD] and 990 vedolizumab-treated patients from the maintenance phase [407 with UC and 583 CD]. Baseline demographics and disease characteristics, summarized using descriptive statistics for the induction and maintenance trial phases [Table 1], were generally comparable between patients receiving vedolizumab with or without 5-ASA. Fewer patients with IBD [UC or CD] receiving 5-ASA co-treatment were anti-TNFα treatment-experienced compared with those treated without 5-ASA [39.6% vs 61.9%, respectively, at induction; 37.2% vs 66.9%, respectively, for the maintenance phase]. This difference was consistently observed in the individual UC and CD disease groups and was more pronounced in patients with CD [37.2% of patients with CD treated with 5-ASA during vedolizumab maintenance had a history of prior anti-TNFα treatment vs 72.2% of patients treated without 5-ASA]. The median [range] duration of disease was shorter in patients with CD receiving vedolizumab maintenance treatment with concomitant 5-ASA (5.84 [0–34] years) compared with 8.0 [0–47] years in patients not receiving 5-ASA (5.8 [0–42] vs 8.3 [0–51] years for patients with CD receiving vedolizumab induction with and without 5-ASA, respectively). The proportion of smokers was similar, regardless of 5-ASA co-treatment status for each disease; approximately one in five patients with CD were current smokers, a higher rate than in patients with UC [Table 1].

**Table 1.** Baseline demographics and disease characteristics [efficacy analysis] for [A] the induction phase and [B] the maintenance phase

A						
Induction phase	Patients with UC		Patients with CD		All patients [UC and CD]	
	With 5-ASA [*n* = 328]	No 5-ASA [*n* = 113]	With 5-ASA [*n* = 290]	No 5-ASA [*n* = 339]	With 5-ASA [*n* = 618]	No 5-ASA [*n* = 452]
Mean [SD] age, years	38.7 [12.8]	42.5 [13.5]	35.4 [12.2]	38.5 [13.4]	37.2 [12.6]	39.5 [13.5]
Male, *n* [%]	196 [59.8]	66 [58.4]	166 [57.2]	162 [47.8]	362 [58.6]	228 [50.4]
Race, white, *n* [%]	266 [81.1]	98 [86.7]	248 [85.5]	308 [90.6]	514 [83.2]	406 [89.8]
Mean [SD] body weight, kg	71.9 [17.0]	76.0 [19.2]	67.7 [18.9]	73.3 [18.8]	69.9 [18.0]	74.0 [18.9]
Current smoker, *n* [%]	27 [8.2]	6 [5.3]	55 [19.0]	79 [23.3]	82 [13.3]	85 [18.8]
Duration of disease, *n*	328	113	290	338	618	451
Mean [SD], years	6.9 [5.8]	7.1 [5.8]	7.3 [6.7]	10.6 [9.1]	7.1 [6.3]	9.8 [8.6]
Mean [SD] Mayo score	8.7 [1.7]	8.8 [1.5]	NA	NA	NA	NA
Mean [SD] CDAI score	NA	NA	320.3 [62.6]	323.8 [62.5]^a^	NA	NA
Faecal calprotectin, *n*	313	110	287	329	600	439
Mean [SD], µg/g	2401.7 [3389.9]	3244.3 [4186.9]	1540.1 [2212.3]	1497.1 [2037.5]	1989.6 [2916.8]	1989.6 [2916.8]
Concomitant medication, *n* [%]	328 [100.0]	109 [96.5]	290 [100.0]	302 [89.1]	618 [100.0]	411 [90.9]
Immunosuppressants only	50 [15.2]	15 [13.3]	66 [22.8]	56 [16.5]	116 [18.8]	71 [15.7]
Corticosteroids only	99 [30.2]	37 [32.7]	81 [27.9]	81 [23.9]	180 [29.1]	118 [26.1]
Corticosteroids and immunosuppressants	67 [20.4]	13 [11.5]	47 [16.2]	35 [10.3]	114 [18.4]	48 [10.6]
No corticosteroids or immunosuppressants	112 [34.1]	48 [42.5]	96 [33.1]	167 [49.3]	208 [33.7]	215 [47.6]
Prior anti-TNFα treatment history, *n* [%]	120 [36.6]	55 [48.7]	125 [43.1]	225 [66.4]	245 [39.6]	280 [61.9]
**B**						
Maintenance phase	Patients with UC		Patients with CD		All patients [UC and CD]	
	With 5-ASA [*n* = 306]	No 5-ASA [*n* = 101]	With 5-ASA [*n* = 234]	No 5-ASA [*n* = 349]	With 5-ASA [*n* = 540]	No 5-ASA [*n* = 450]
Mean [SD] age, years	39.3 [13.3]	40.6 [14.2]	35.8 [12.8]	37.0 [13.3]	37.8 [13.2]	37.8 [13.6]
Male, *n* [%]	181 [59.2]	53 [52.5]	136 [58.1]	171 [49.0]	317 [58.7]	224 [49.8]
Race, white, *n* [%]	260 [85.0]	84 [83.2]	202 [86.3]	318 [91.1]	462 [85.6]	402 [89.3]
Mean [SD] body weight, kg	74.4 [17.9]	74.1 [17.0]	67.9 [17.1]	74.6 [19.5]	71.6 [17.9]	74.5 [18.9]
Current smoker, *n* [%]	28 [9.2]	8 [7.9]	48 [20.5]	93 [26.6]	76 [14.1]	101 [22.4]
Duration of disease, *n*	306	101	234	348	540	449
Mean [SD], years	7.3 [5.9]	7.5 [6.6]	6.9 [6.0]	10.0 [8.4]	7.2 [6.0]	9.4 [8.1]
Mean [SD] Mayo score	8.6 [1.6]	8.5 [1.7]	NA	NA	NA	NA
CDAI score, *n*	NA	NA	233	348	NA	NA
Mean [SD]	NA	NA	322.5 [64.4]	319.4 [62.0]	NA	NA
Faecal calprotectin, *n*	293	95	230	340	523	435
Mean [SD], µg/g	1997.0 [3005.6]	2649.6 [3644.1]	1235.7 [1767.7]	1205.0 [1608.0]	1662.2 [2562.6]	1520.5 [2291.9]
Concomitant medication, *n* [%]	306 [100.0]	96 [95.0]	234 [100.0]	321 [92.0]	540 [100.0]	417 [92.7]
Immunosuppressants only	54 [17.6]	15 [14.9]	51 [21.8]	58 [16.6]	105 [19.4]	73 [16.2]
Corticosteroids only	108 [35.3]	29 [28.7]	78 [33.3]	103 [29.5]	186 [34.4]	132 [29.3]
Corticosteroids and immunosuppressants	58 [19.0]	14 [13.9]	34 [14.5]	42 [12.0]	92 [17.0]	56 [12.4]
No corticosteroids or immunosuppressants	86 [28.1]	43 [42.6]	71 [30.3]	146 [41.8]	157 [29.1]	189 [42.0]
Prior anti-TNFα treatment history, *n* [%]	114 [37.3]	49 [48.5]	87 [37.2]	252 [72.2]	201 [37.2]	301 [66.9]

5-ASA, 5-aminosalicylic acid; CD, Crohn’s disease; CDAI, Crohn’s disease activity index; NA, not applicable; SD, standard deviation; TNF, tumour necrosis factor; UC, ulcerative colitis.

Data are from four clinical trials [GEMINI 1, GEMINI 2, VISIBLE 1, and VISIBLE 2].

^a^ *n* = 338.

### 3.2. Efficacy

#### 3.2.1. Patients with UC

At Week 6, a clinical response was achieved by 246 of 328 [75.0%] and 70 of 113 [61.9%] patients with UC receiving vedolizumab induction with and without 5-ASA co-treatment, respectively. Week 6 clinical remission rates were 68 of 328 [20.7%] and 23 of 113 [20.4%] patients in these two treatment groups, respectively, increasing after re-randomization of responders to 138 of 306 [45.1%] and 41 of 101 [40.6%] by Week 52. Rates of CS-free clinical remission at Week 52 were 57 of 166 [34.3%] patients for vedolizumab and 5-ASA co-treatment and 17 of 43 [39.5%] patients for vedolizumab treatment without 5-ASA.

**Figure 2.**
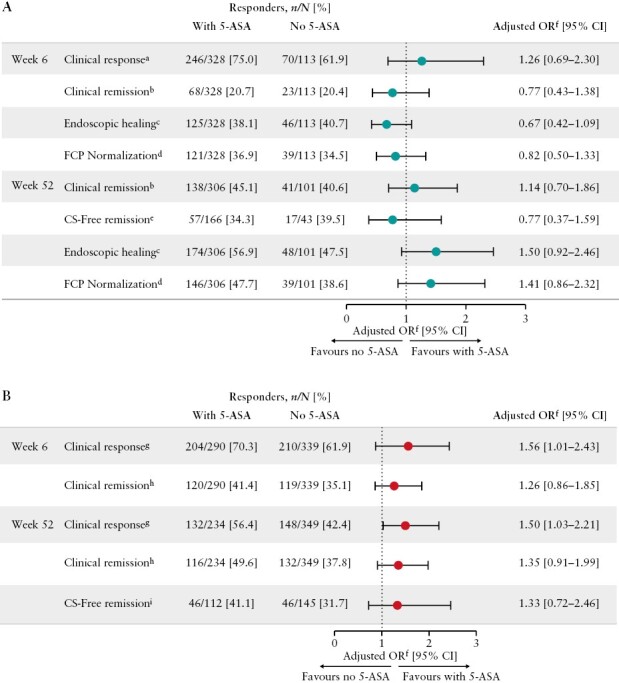
Adjusted ORs of patients with inflammatory bowel disease achieving efficacy outcomes following induction [Week 6] with vedolizumab IV and maintenance [Week 52] with vedolizumab IV or SC, with vs without 5-ASA in [A] patients with UC and [B] patients with CD. Data on patients with UC are from the GEMINI 1 and VISIBLE 1 studies. Data on patients with CD are from the GEMINI 2 and VISIBLE 2 studies. Patients with missing data were imputed as not having achieved the efficacy outcome [i.e. non-responders, non-remitters]. 5-ASA, 5-aminosalicylic acid; CD, Crohn’s disease; CDAI, Crohn’s disease activity index; CI, confidence interval; CS, corticosteroids; FCP, faecal calprotectin; IV, intravenous; OR, odds ratio; SC, subcutaneous; UC, ulcerative colitis. ^a^Clinical response in patients with UC was defined as a reduction in complete Mayo score of ≥3 and ≥30% from baseline with an accompanying decrease in rectal bleeding subscore of ≥1 or absolute rectal bleeding subscore of ≤1. ^b^Clinical remission in patients with UC was defined as a complete Mayo score of ≤2 points and no individual subscore of >1 point. ^c^Endoscopic healing in patients with UC was defined as Mayo endoscopic subscore of ≤1 point. ^d^FCP normalization in patients with UC was defined as FCP ≤250 µg/g. ^e^CS-free clinical remission at Week 52 in patients with UC was defined as a complete Mayo score of ≤2 points and no individual subscore of >1 point achieved in patients who were receiving oral CS at baseline [Week 0] and had discontinued oral CS at Week 52. ^f^ORs are adjusted for age, sex, concomitant steroid use at baseline [dose of prednisone], baseline immunomodulator use, disease localization, smoking status, baseline albumin, baseline FCP, prior anti-tumour necrosis factor-α treatment experience, and baseline Mayo endoscopy subscore [UC only]. ^g^Clinical response was defined as a ≥100-point decrease in CDAI score from baseline in patients with CD. ^h^Clinical remission was defined as a CDAI score of ≤150 in patients with CD. ^i^CS-free clinical remission at Week 52 was defined as patients with CD with a CDAI score of ≤150 who were receiving oral CS at baseline [Week 0] and had discontinued oral CS at Week 52.

Based on the multivariate analysis of vedolizumab-treated patients with UC [Figure 2A], there was no impact of concomitant 5-ASA on clinical response or remission at Week 6 or clinical remission and CS-free remission at Week 52. The proportion of patients with endoscopic healing [Mayo endoscopic subscore of ≤1 point] after vedolizumab induction at Week 6 was 125 of 328 [38.1%] in the group receiving 5-ASA co-treatment, and was similar to the group treated without 5-ASA (46 of 113 [40.7%]) with an aOR of 0.67 [95% CI: 0.42–1.09]. By Week 52, the proportion of patients with endoscopic healing was numerically higher for vedolizumab treatment with 5-ASA (174 of 306 [56.9%]) vs without 5-ASA (48 of 101 [47.5%]) but no significant differences were seen on adjusted analyses (aOR 1.50 [95% CI: 0.92–2.46]) [Figure 2A]. FCP normalization [FCP ≤250 µg/g] after vedolizumab induction and maintenance phases followed a similar pattern to the data for endoscopic healing, with no significant differences related to 5-ASA co-treatment observed [Figure 2A].

#### 3.2.2. Patients with CD

The proportion of patients with CD achieving clinical remission or response by Week 6 was similar in patients receiving vedolizumab with or without 5-ASA. Week 6 clinical remission rates were 120 of 290 [41.4%] and 119 of 339 [35.1%] patients in treatment groups with and without 5-ASA, respectively, and rates of clinical response were 204 of 290 [70.3%] and 210 of 339 [61.9%], respectively [Figure 2B]. At Week 52, the proportion of vedolizumab-treated patients with CD achieving clinical remission was 116 of 234 [49.6%] patients receiving concomitant 5-ASA, compared with 132 of 349 [37.8%] in the group treated without 5-ASA. In addition, 46 of 112 [41.1%] patients co-treated with 5-ASA achieved CS-free remission, compared with 46 of 145 [31.7%] patients not receiving 5-ASA. Week 52 rates of response were 56.4% [132 of 234] with 5-ASA and 42.4% [148 of 349] without 5-ASA (aOR 1.50 [95% CI: 1.03-2.21]); [Figure 2B].

Considering efficacy outcomes analysed in patients with CD overall, although aORs for clinical response marginally favoured concomitant 5-ASA use, most efficacy outcomes analysed failed to show a clear benefit for concomitant 5-ASA treatment [Figure 2B].

**Figure 3.**
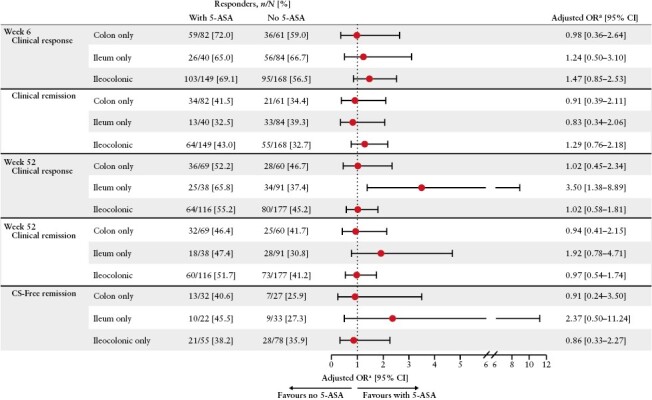
Adjusted ORs of patients with CD achieving efficacy outcomes by baseline disease location, with vs without 5-ASA. Data on patients with CD are from the GEMINI 2 and VISIBLE 2 studies. Patients with missing data were imputed as not having achieved the efficacy outcome [i.e. non-responders, non-remitters]. 5-ASA, 5-aminosalicylic acid; CD, Crohn’s disease; CI, confidence interval; CS, corticosteroids; OR, odds ratio. ^a^ORs are adjusted for age, sex, concomitant steroid use at baseline [dose of prednisone], baseline immunomodulator use, disease localization, smoking status, baseline albumin, baseline faecal calprotectin, and prior anti-tumour necrosis factor-α treatment experience.

Rates of clinical remission and response achieved after vedolizumab induction at Week 6 analysed by baseline disease location were also similar, regardless of 5-ASA co-treatment in patients with colon-only, ileum-only or ileocolonic disease [Figure 3]. Clinical response at Week 52 for patients with ileal disease was the only outcome from the subgroup analysis by disease location where the aOR favoured treatment with 5-ASA, albeit with a wide confidence interval. There were no differences with regard to 5-ASA treatment for clinical remission at Week 52 analysed by disease location [Figure 3].

### 3.3. Safety/tolerability in patients with UC or CD

Pooled safety data were analysed from patients with IBD [patients with UC and CD combined] in four clinical trials of vedolizumab IV treatment [1279 patients treated with concomitant 5-ASA and 1052 without] and three trials of vedolizumab SC [484 patients treated with 5-ASA and 327 without] [Table 2].

**Table 2.** Overview of AEs

Variable	Patients with UC		Patients with CD		All patients [UC and CD]	
	With 5-ASA	No 5-ASA	With 5-ASA	No 5-ASA	With 5-ASA	No 5-ASA
Vedolizumab IV^a^						
* n*	674	184	605	868	1279	1052
** AEs, *n* [%]**	622 [92.3]	172 [93.5]	566 [93.6]	832 [95.9]	1188 [92.9]	1004 [95.4]
** Not treatment related**	326 [48.4]	87 [47.3]	349 [57.7]	352 [40.6]	675 [52.8]	439 [41.7]
** Treatment related**	296 [43.9]	85 [46.2]	217 [35.9]	480 [55.3]	513 [40.1]	565 [53.7]
** Mild**	117 [17.4]	27 [14.7]	101 [16.7]	109 [12.6]	218 [17.0]	136 [12.9]
** Moderate**	348 [51.6]	88 [47.8]	304 [50.2]	400 [46.1]	652 [51.0]	488 [46.4]
** Severe**	157 [23.3]	57 [31.0]	160 [26.4]	323 [37.2]	317 [24.8]	380 [36.1]
** Leading to study discontinuation**	118 [17.5]	29 [15.8]	111 [18.3]	173 [19.9]	229 [17.9]	202 [19.2]
** Serious AEs, *n* [%]**	221 [32.8]	69 [37.5]	240 [39.7]	390 [44.9]	461 [36.0]	459 [43.6]
** Not treatment related**	190 [28.2]	56 [30.4]	220 [36.4]	311 [35.8]	410 [32.1]	367 [34.9]
** Treatment related**	31 [4.6]	13 [7.1]	20 [3.3]	79 [9.1]	51 [4.0]	92 [8.7]
** Leading to study discontinuation**	54 [8.0]	15 [8.2]	68 [11.2]	108 [12.4]	122 [9.5]	123 [11.7]
** Death, *n* [%]**	0 [0.0]	3 [1.6]	2 [0.3]	8 [0.9]	2 [0.2]	11 [1.0]
Vedolizumab SC^b^						
* n*	245	59	239	268	484	327
** AEs, *n* [%]**	173 [70.6]	45 [76.3]	177 [74.1]	207 [77.2]	350 [72.3]	252 [77.1]
** Not treatment related**	122 [49.8]	27 [45.8]	131 [54.8]	144 [53.7]	253 [52.3]	171 [52.3]
** Treatment related**	51 [20.8]	18 [30.5]	46 [19.2]	63 [23.5]	97 [20.0]	81 [24.8]
** Mild**	73 [29.8]	23 [39.0]	77 [32.2]	66 [24.6]	150 [31.0]	89 [27.2]
** Moderate**	82 [33.5]	16 [27.1]	78 [32.6]	110 [41.0]	160 [33.1]	126 [38.5]
** Severe**	18 [7.3]	6 [10.2]	22 [9.2]	31 [11.6]	40 [8.3]	37 [11.3]
** Leading to study discontinuation**	13 [5.3]	4 [6.8]	11 [4.6]	14 [5.2]	24 [5.0]	18 [5.5]
** Serious AEs, *n* [%]**	37 [15.1]	9 [15.3]	44 [18.4]	44 [16.4]	81 [16.7]	53 [16.2]
** Not treatment related**	34 [13.9]	8 [13.6]	36 [15.1]	41 [15.3]	70 [14.5]	49 [15.0]
** Treatment related**	3 [1.2]	1 [1.7]	8 [3.3]	3 [1.1]	11 [2.3]	4 [1.2]
** Leading to study discontinuation**	9 [3.7]	2 [3.4]	4 [1.7]	5 [1.9]	13 [2.7]	7 [2.1]
** Death, *n* [%]**	0 [0.0]	0 [0.0]	0 [0.0]	1 [0.4]	0 [0.0]	1 [0.3]

AEs summarized were reported after the first IV or SC dose of vedolizumab and within 126 days of the last IV or SC dose.

5-ASA, 5-aminosalicylic acid; AE, adverse event; CD, Crohn’s disease; IV, intravenous; SC, subcutaneous; UC, ulcerative colitis.

^a^Data are from four clinical trials [GEMINI 1, GEMINI 2, GEMINI 3, and GEMINI long-term safety study].

^b^Data are from three clinical trials [VISIBLE 1, VISIBLE 2, and VISIBLE open-label extension].

The proportion of patients with IBD experiencing AEs overall was comparable between groups treated with or without 5-ASA; proportions were also similar, irrespective of 5-ASA co-treatment, across different AE subgroups [categorized by AE severity, treatment-related AEs or AEs leading to discontinuation] [Table 2; Supplementary Table 1]. For the vedolizumab IV studies, overall, <9% of patients with IBD experienced serious treatment-related AEs [4.0% in the concomitant 5-ASA group and 8.7% of patients treated without 5-ASA], and for the vedolizumab SC studies, <3% of patients had serious treatment-related AEs [2.3% for treatment with 5-ASA and 1.2% for treatment without 5-ASA]. For deaths reported within 126 days of the last vedolizumab IV dose, two occurred in the concomitant 5-ASA group and 11 in the without 5-ASA group. No deaths were reported within 126 days of the last SC dose in the group receiving 5-ASA; one was reported in the without 5-ASA group.

**Figure 4.**
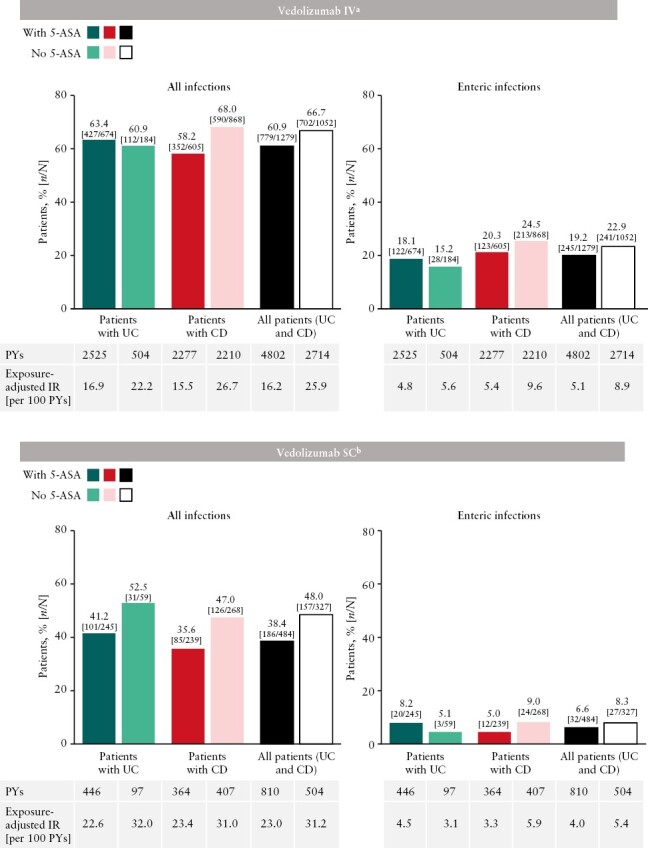
Incidence rates of all infections and enteric infections in patients treated with vedolizumab IV or SC by concomitant 5-ASA treatment status. Adverse events were reported after the first IV or SC dose of vedolizumab and within 126 days of the last IV or SC dose. 5-ASA, 5-aminosalicylic acid; CD, Crohn’s disease; IR, incidence rate; IV, intravenous; PY, patient-year; SC, subcutaneous; UC, ulcerative colitis. ^a^Data are from four vedolizumab IV clinical trials [GEMINI 1, GEMINI 2, GEMINI 3, and GEMINI long-term safety]. ^b^Data are from three vedolizumab SC clinical trials [VISIBLE 1, VISIBLE 2, and VISIBLE open-label extension].

The incidence of enteric infections [exposure-adjusted incidence per 100 PYs] was low in patients with IBD receiving vedolizumab IV/SC: 5.1 for vedolizumab IV treatment with 5-ASA and 8.9 without 5-ASA [Figure 4]. Overall incidence rates of all infections [exposure-adjusted incidence per 100 PYs] were 16.2 and 25.9 for vedolizumab IV treatment with and without 5-ASA, respectively [Figure 4; Supplementary Table 1]. There was little difference between the incidence rates of enteric infections or all infections by 5-ASA co-treatment status when analysed only in patients receiving concomitant CS or IMMs in vedolizumab IV/SC studies [Supplementary Table 1]. The incidence rate of serious treatment-related GI infections was low [≤0.1 per 100 PYs] in patients who received vedolizumab IV with or without 5-ASA. No treatment-related serious GI infections were reported in patients who received vedolizumab SC, irrespective of 5-ASA treatment.

## 4. Discussion

In this *post hoc* analysis of pooled data from phase 3 and OLE vedolizumab clinical trials, we observed that concomitant 5-ASA treatment has no impact on the overall efficacy and safety of vedolizumab treatment in patients with moderate to severe IBD. In the multivariate analysis, the odds of achieving clinical remission and steroid-free remission at Weeks 6 and 52 [adjusted for variables including prior anti-TNFα treatment] were not significantly different with or without 5-ASA among vedolizumab-treated patients. There was also no clear signal on concomitant 5-ASA treatment for clinical response and secondary outcomes of endoscopic healing and FCP normalization, analysed in patients with IBD. There was a weak signal of improved clinical response with 5-ASA at Week 52 for patients with ileal CD, but with a wide confidence interval. Clinical response is not a targeted maintenance endpoint in patients with CD.

The safety profile of vedolizumab IV and SC, with and without 5-ASA, was consistent with the established safety profile of vedolizumab. There was no evidence from this analysis that 5-ASA treatment had any impact on the incidence of AEs in patients with IBD treated with vedolizumab IV or SC. No treatment-related serious GI infections were observed with vedolizumab SC, either with or without 5-ASA, and the incidence rate of treatment-related serious GI infections with vedolizumab IV with or without 5-ASA was low [≤0.1 per 100 PYs].

Current evidence on the benefit–risk of combination therapy with a biologic and 5-ASA is limited.^16,22,24–28^ Several *post hoc* clinical trial analyses and studies of observational health claims data have, so far, not indicated a clear benefit for concomitant 5-ASA treatment in terms of clinical effectiveness and/or safety outcomes in patients escalated to anti-TNFα treatment. In a *post hoc* analysis of patient-level data, concomitant use of 5-ASA was not associated with higher rates of clinical remission, response or mucosal healing (from a multivariable regression analysis of pooled data from patients with moderate to severe UC from five clinical trials [*n* = 2183] receiving infliximab or golimumab).^26^ 5-ASA co-treatment was associated with a reduced likelihood of achieving remission in adalimumab-treated patients with severely active UC [Mayo score of ≥10].^24^ A retrospective cohort study of real-world data from two large US and Danish healthcare databases reported that stopping 5-ASA after initiation of anti-TNFα treatment was not associated with an increased risk of adverse clinical outcomes [new steroid use, UC-related hospitalization or surgery] vs continuing 5-ASA. The data set included patients with UC receiving oral 5-ASA treatment for ≥90 days [*n* = 3589].^27^ A parallel study of patients with CD receiving oral mesalamine for ≥90 days from the same two databases [*n* = 3178] also showed no worse clinical events associated with stopping vs continuing mesalamine when escalating to anti-TNFα treatment.^28^ A retrospective study of 1403 patients in Hong Kong with stable UC not on biologic treatments (in corticosteroid-free remission for at least 1 year) reported that stopping 5-ASA was not associated with an increased risk of disease flare (adjusted hazard ratio 0.91 [95% CI: 0.64–1.31]; *p* = 0.620) over a median 41.8 months of follow-up.^22^ Although 5-ASA might seem inexpensive relative to biologics, cumulative use can pose a significant financial burden.^26^ In a cost-effectiveness analysis of patients with UC who received biologics or tofacitinib, continuing 5-ASA therapy was not considered to be a cost-effective strategy.^39^ 5-ASA co-treatment may represent low-value care if there is no evidence of additional efficacy resulting from continued 5-ASA use.^26^

Other observational data can be supportive of the effectiveness of 5-ASA when included as a combination therapy in UC.^40^ A cohort study of patients with UC in clinical remission reported a reduced risk of disease flare in patients adherent to 5-ASA therapy.^41^ Data from a nationwide Swiss IBD cohort study suggest that adding 5-ASA to a biologic may lower the risk of colonic surgery in patients with UC.^25^ A possible reason for continued use of 5-ASA in UC and CD, even after treatment failure, may be the perceived chemoprotective effects of 5-ASA treatment. This is based on some studies reporting reduction of colorectal cancer risk with 5-ASA treatment ^18^^,^^19,20^; however, it is unclear if this effect is specific to 5-ASA treatment or mucosal healing in general.^17^

Emerging evidence suggests that withdrawal of 5-ASA can be safe in patients with CD and in patients with UC who are in deep remission and do not have other risk factors, such as extensive disease or history of multiple flares.^16^ It has been suggested that 5-ASA withdrawal should be considered in all patients with CD and on an individual basis in patients with UC who achieved remission with biologic treatment.^16^

Several limitations to note for this *post hoc* analysis are the inclusion of data from open-label treatment and the low patient numbers in some subgroups analysed [notably for safety data in the UC population and infection data on vedolizumab SC treatment]. In addition, given the *post hoc* nature of the analysis of efficacy data, no multiplicity adjustment was performed. The VARSITY study, in which vedolizumab was compared with adalimumab in adults with moderate to severe UC, was not included in the safety analysis because of the difficulties associated with pooling integrated safety data.^42^ Potential confounding factors not accounted for in the multivariate analysis include histopathological differences at baseline. Also, patient selection criteria required for inclusion in clinical trials have limiting effects on the generalizability of findings to patients treated in real-world clinical settings.^43^ The comparison of outcomes for vedolizumab treatment in patients receiving concomitant 5-ASA, at any time during the studies, does not directly assess the benefit–risk of stopping 5-ASA at the time of escalation to biologic therapy.

In conclusion, concomitant 5-ASA does not appear to impact the efficacy of vedolizumab IV or SC treatment in patients with IBD. Although there were limited data for some subgroups, there was no evidence to suggest that concomitant 5-ASA use impacted AE rates. These data will be useful to inform benefit–risk assessments of concomitant 5-ASA treatment in patients initiating vedolizumab therapy. Prospective interventional trials of 5-ASA withdrawal at the time of escalation to advanced therapy are warranted to validate findings from this and other retrospective analyses, and to confirm that patients with IBD who require escalation to advanced therapy can safely discontinue 5-ASA treatment.

## Supplementary Material

jjad113_suppl_Supplementary_Material

## Data Availability

The datasets, including the redacted study protocol, redacted statistical analysis plan, and individual participants’ data supporting the results reported in this article will be made available within 3 months from initial request, to researchers who provide a methodologically sound proposal. The data will be provided after its de-identification, in compliance with applicable privacy laws, data protection, and requirements for consent and anonymization. Data are available on request via application at https://search.vivli.org/.

## Conference Presentation

Poster presentation P290 at the 16th Congress of the European Crohn’s and Colitis Organisation, July 2–3 & 8–10, 2021, Virtual congress [*J Crohns Colitis* 15(Suppl. 1): S321].
