# Supplementary material for: Impact of Concomitant 5-Aminosalicylic Acid Therapy on Vedolizumab Efficacy and Safety in Inflammatory Bowel Disease: Post Hoc Analyses of Clinical Trial Data
Source: J Crohns Colitis. 2023 Jul 26;17(12):1949–61. doi: 10.1093/ecco-jcc/jjad113 (PMC10798864; doi:10.1093/ecco-jcc/jjad113)
Supplement: jjad113_suppl_Supplementary_Material [file jjad113_suppl_supplementary_material.docx]

**Supplementary Data**

**Supplementary Table 1.** All infections and enteric infections in patients with UC or CD who received vedolizumab IV or SC by 5-ASA concomitant treatment status

|  | **Variable** | **Patients with UC** | | **Patients with CD** | | **All patients [UC and CD]** | |
| --- | --- | --- | --- | --- | --- | --- | --- |
|  |  | **With 5-ASA** | **No 5-ASA** | **With 5-ASA** | **No 5-ASA** | **With 5-ASA** | **No 5-ASA** |
|  | Vedolizumab IV,^a^ *n* | 674 | 184 | 605 | 868 | 1279 | 1052 |
|  | Patient-years | 2525 | 504 | 2277 | 2210 | 4802 | 2714 |
|  | All infections, *n* [%] | 427 [63.4] | 112 [60.9] | 352 [58.2] | 590 [68.0] | 779 [60.9] | 702 [66.7] |
|  | Exposure-adjusted incidence rate  [per 100 patient-years] | 16.9 | 22.2 | 15.5 | 26.7 | 16.2 | 25.9 |
|  | Enteric infections, *n* [%] | 122 [18.1] | 28 [15.2] | 123 [20.3] | 213 [24.5] | 245 [19.2] | 241 [22.9] |
|  | Exposure-adjusted incidence rate  [per 100 patient-years] | 4.8 | 5.6 | 5.4 | 9.6 | 5.1 | 8.9 |
| With IMM or CS | Vedolizumab IV,^a^ *n* | 674 | 182 | 590 | 826 | 1264 | 1008 |
|  | Patient-years | 2525 | 497 | 2229 | 2129 | 4754 | 2625 |
|  | All infections, *n* [%] | 427 [63.4] | 111 [61.0] | 345 [58.5] | 566 [68.5] | 772 [61.1] | 677 [67.2] |
|  | Exposure-adjusted incidence rate  [per 100 patient-years] | 16.9 | 22.3 | 15.5 | 26.6 | 16.2 | 25.8 |
|  | Enteric infections, *n* [%] | 122 [18.1] | 28 [15.4] | 120 [20.3] | 202 [24.5] | 242 [19.1] | 230 [22.8] |
|  | Exposure-adjusted incidence rate  [per 100 patient-years] | 4.8 | 5.6 | 5.4 | 9.5 | 5.1 | 8.8 |
|  | Vedolizumab SC,^b^ *n* | 245 | 59 | 239 | 268 | 484 | 327 |
|  | Patient-years | 446 | 97 | 346 | 407 | 810 | 504 |
|  | All infections, *n* [%] | 101 [41.2] | 31 [52.5] | 85 [35.6] | 126 [47.0] | 186 [38.4] | 157 [48.0] |
|  | Exposure-adjusted incidence rate [per 100 patient-years] | 22.6 | 32.0 | 23.4 | 31.0 | 23.0 | 31.2 |
|  | Enteric infections, *n* [%] | 20 [8.2] | 3 [5.1] | 12 [5.0] | 24 [9.0] | 32 [6.6] | 27 [8.3] |
|  | Exposure-adjusted incidence rate [per 100 patient-years] | 4.5 | 3.1 | 3.3 | 5.9 | 4.0 | 5.4 |
| With IMM or CS | Vedolizumab SC,^b^ *n* | 168 | 40 | 174 | 154 | 342 | 194 |
|  | Patient-years | 311 | 62 | 264 | 235 | 574 | 298 |
|  | All infections, *n* [%] | 82 [48.8] | 20 [50.0] | 68 [39.1] | 76 [49.4] | 150 [43.9] | 96 [49.5] |
|  | Exposure-adjusted incidence rate [per 100 patient-years] | 26.4 | 32.3 | 25.8 | 32.3 | 26.1 | 32.2 |
|  | Enteric infections, *n* [%] | 17 [10.1] | 1 [2.5] | 9 [5.2] | 15 [9.7] | 26 [7.6] | 16 [8.2] |
|  | Exposure-adjusted incidence rate [per 100 patient-years] | 5.5 | 1.6 | 3.4 | 6.4 | 4.5 | 5.4 |

5-ASA, 5-aminosalicylic acid; CD, Crohn’s disease, CS, corticosteroids; IMM, immunomodulator; IV, intravenous; SC, subcutaneous; UC, ulcerative colitis.

^a^Data are from four clinical trials [GEMINI 1, GEMINI 2, GEMINI 3 and GEMINI long-term safety].

^b^Data are from three clinical trials [VISIBLE 1, VISIBLE 2 and VISIBLE open-label extension].
